# Pseudo-pseudo Meigs syndrome in a patient with overlap syndrome: A case report and literature review

**DOI:** 10.1097/MD.0000000000044857

**Published:** 2025-10-03

**Authors:** Hao Zhang, Jintao Zhang, Ningning Li, Bingbing Dai

**Affiliations:** a Department of Rheumatology and Immunology, Central Hospital of Dalian University of Technology, Dalian, China.

**Keywords:** case report, overlap syndrome, Pseudo-pseudo Meigs syndrome, systemic lupus erythematosus, systemic sclerosis

## Abstract

**Rationale:**

Pseudo-pseudo Meigs syndrome (PPMS), characterized by pleural effusion, ascites, and elevated cancer antigen-125 (CA-125) levels, is a rare complication of autoimmune diseases, particularly of systemic lupus erythematosus-systemic sclerosis (SLE-SSc) overlap syndrome.

**Patient concerns:**

A 39-year-old female presented with progressive symptoms, including finger swelling (since 13 years), skin hardening (since 10 years), bilateral second toe ulceration (since 3 months), and abdominal distention (since 1 month). During treatment for pulmonary arterial hypertension (PAH), she developed significant ascites with elevated CA-125 levels.

**Diagnoses:**

Comprehensive clinical evaluation and laboratory tests confirmed SLE-SSc overlap syndrome. Subsequently, contrast-enhanced abdominal magnetic resonance imaging and multidisciplinary consultation established the diagnosis of PPMS, attributed to both active disease and possible medication effects.

**Interventions:**

Management included optimization of immunosuppressive therapy and discontinuation of PAH medications (Sildenafil and Ambrisentan).

**Outcomes:**

Complete resolution of ascites was achieved, and the patient maintains stable condition under regular outpatient follow-up.

**Lessons:**

This case emphasizes the importance of considering PPMS in autoimmune disease patients with unexplained ascites and elevated CA-125 levels, while suggesting a potential association between PAH medications and PPMS development.

## 1. Introduction

Meigs syndrome is a distinct clinical entity characterized by the presence of ascites, pleural effusion, and one of the 4 types of benign ovarian tumors: fibroma, thecoma, Brenner tumor, or the rare granulosa cell tumor.^[1]^ A related condition, pseudo-Meigs syndrome, presents with similar manifestations including ascites and pleural effusion but is associated with pelvic tumors other than ovarian fibroma, including struma ovarii, colorectal cancer, and uterine leiomyoma.^[2]^

In patients with systemic lupus erythematosus (SLE), a unique presentation known as pseudo-pseudo Meigs syndrome (PPMS) or Tjalma syndrome has been described. This condition is characterized by massive ascites, pleural effusion, and markedly elevated serum cancer antigen (CA)-125 levels in the absence of any ovarian tumor.^[3]^

Here, we present a rare case of PPMS in a patient with overlap syndrome of SLE and systemic sclerosis (SSc). This case highlights the importance of considering PPMS in the differential diagnosis of patients with autoimmune diseases who present with unexplained ascites and elevated CA-125 levels. Through this case report and literature review, we aim to enhance the understanding of this uncommon complication and facilitate its timely diagnosis and appropriate management.

## 2. Clinical presentation

### 2.1. Previous medical history

A 39-year-old female presented to our department with a 13-year history of finger swelling, 10-year history of skin hardening, 3-month history of bilateral second toe ulceration, and 1-month history of abdominal distention. She had no previous surgical history or any other significant medical conditions.

Her initial symptoms began 13 years ago with painful finger swelling. Initial laboratory tests revealed a positive antinuclear antibody (ANA) titer of 1:3200, leading to a diagnosis of connective tissue disease treated with corticosteroids. Ten years ago, she developed progressive skin hardening and was diagnosed with SSc at a tertiary hospital. Treatment included corticosteroids (maximum prednisone dose 40 mg/d) and cyclophosphamide (cumulative dose 5.6 g). During this period, she experienced gastrointestinal involvement manifesting as dysphagia, acid reflux, and bloating, and gastroscopy revealed a watermelon stomach.

Three years ago, she was diagnosed with overlap syndrome (SSc and SLE) based on pancytopenia and positive anti-dsDNA antibodies. One year prior to admission, she developed recurrent palpitations, and echocardiography revealed elevated pulmonary arterial pressure (54 mm Hg in May 2024). She was managed with prednisone 10 mg/d, cyclosporine (100 mg in the morning and 50 mg in the evening), and Sildenafil 25 mg thrice daily.

Three months before admission, she developed bilateral second toe ulceration with exudation. She was hospitalized and treated with methylprednisolone 40 mg/d intravenously for suspected ischemic necrosis. Treatment was escalated to include combination therapy with Sildenafil and Ambrisentan for pulmonary hypertension and digital ulcers. As the toe ulcers improved, mycophenolate mofetil (0.5 g twice daily) was added, and prednisone was gradually tapered to 30 mg/d. One month prior to the current admission, she developed progressive abdominal distention accompanied by intermittent bilateral foot edema.

### 2.2. Physical examination, laboratory, and imaging findings

On admission, her vital signs were as follows: temperature 36.0°C, pulse 80 beats/min, respiratory rate 20 breaths/min, and blood pressure 94/70 mm Hg. Physical examination revealed clear breath sounds bilaterally, regular heart rhythm, abdominal distention with positive shifting dullness, no tenderness or rebound tenderness, and mild bilateral lower extremity edema.

Laboratory investigations showed thrombocytopenia and iron deficiency anemia (detailed values presented in Table 1). Immunological tests revealed positive antinuclear antibodies (IgG) with titers 1:320 to 1000, positive anti-Scl-70 antibodies, and weakly positive anti-Ro-52 antibodies. Antiphospholipid antibody and lupus anticoagulant tests were negative. C-reactive protein was elevated at 13.15 mg/L, complement C3 was decreased to 0.367 g/L (normal range: 0.700–1.400 g/L), and CA-125 was elevated at 92.80 U/mL (normal range: 0 to 47 U/mL).

**Table 1:** Laboratory parameters before and after treatment.

Test	2025/2/28	2025/3/5	2025/3/7	2025/3/25	2025/3/26	2025/4/1	2025/5/9
CA-125 U/mL (0–47 U/ml)	92.8	70.3			70.9	30.4	30.8
Specific gravity (ascitic fluid)	1.014	1.014	1.01	1.012	1.012		
Protein quantification (ascitic fluid)	2.401	2.401	1.029	1.715	1.715		
Total cellular score (ascitic fluid)	4019	2691	610	835	407		
White cell count (ascitic fluid)	215	168	118	120	55		
Neutrophilic granulocyte percentage (ascitic fluid)	7	7	7	10	10		
Percentage of lymphocytes (ascitic fluid)	46	40	61	46	40		
Percentage of monocytes (ascitic fluid)	16	20	6	11	6		
Percentage of mesothelial cells (ascitic fluid)	31	33	26	7			
ADA (ascitic fluid)	2	2	2	2	2		
LDH (ascitic fluid)	47	35	34	59	55		
CEA (ascitic fluid)	0.57	0.62	0.62	0.68	0.53		
CA-125 (ascitic fluid, U/mL [0–47 U/mL])	67.4	65.3			69.4		
Abdominal Circumference (cm)	92	82	80	80	74	72	72
Body weight (kg)	56	54	52.5	50.5	50	47.5	46.7
White blood cell count*10–12/L	2.97	3.17	1.37	3.51	4.78	4.09	6.66
Hemoglobin (g/L)	83	76	78	84	89	93	94
Blood platelet count*10–9/L	20	11	12	22	25	27	27
Complement C3 (0.9–1.8 g/L)	0.367				0.905		
Complement C4 (0.1–0.4 g/L)	0.25				0.24		
ANA							
Titer	1:1000				1:320–1000		
Caryogram	Nuclear particle type				Nuclear particle type		
Anti-dsDNA	210				47.5		
Anti-SM antibody	−				−		
Anti-SSA antibody	±				−		
Anti-SSB antibody	±				−		
Anti-Scl-70 antibody	+				+		
Anti-Ro-52 antibody	±				±		

CA-125 = cancer antigen-125.

Serial ascitic fluid analyses (n = 5) showed yellow, slightly turbid fluid with a specific gravity of 1.010 to 1.014 and protein content of 1.029 to 2.401 g/dL. Cell count analysis revealed a total cell count of 610 to 4019/mm^3^ and white blood cell count of 118 to 215/mm^3^, with differential counts showing a neutrophil count of 7% to 10%, lymphocyte count of 40% to 61%, monocyte count of 6% to 20%, and mesothelial cell count of 26% to 33%. The ascitic fluid CA-125 level was 70.90 U/mL. Cytological examination of the ascitic fluid showed lymphocytes and mesothelial cells without malignant cells.

**Figure 1.**
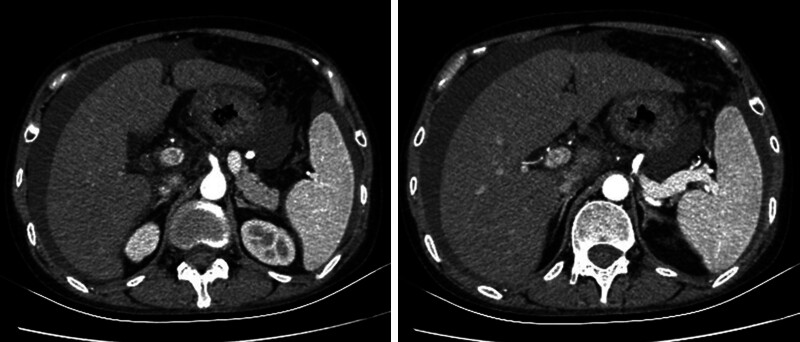
Contrast-enhanced abdominal CT showing ascites and portal vein dilatation without evident portal vein thrombosis. CT = computed tomography.

**Figure 2.**
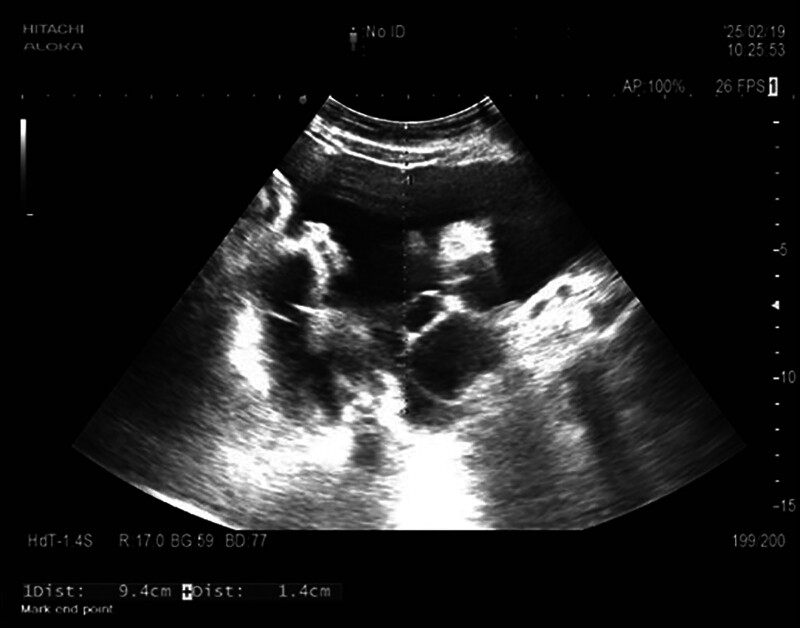
Abdominal ultrasound demonstrating ascites with a maximum depth of approximately 9.4 cm.

Imaging studies included high-resolution chest computed tomography, which revealed bilateral interstitial changes, new-onset bilateral pleural effusions, and distal esophageal wall thickening with proximal dilation. Abdominal contrast-enhanced computed tomography demonstrated mild hepatic density reduction, portal vein dilation, gallbladder wall thickening with bile stasis, multiple small retroperitoneal lymph nodes, and massive ascites (Fig. 1). Enhanced abdominal magnetic resonance imaging showed portal vein dilation with splenorenal shunting and portal vein flow velocity of 32 cm/s with branch velocity of 17 cm/s. Echocardiography estimated pulmonary arterial systolic pressure at 59 mm Hg with mild tricuspid regurgitation and normal left ventricular function. Lower extremity vascular ultrasound was unremarkable. Ultrasound confirmed small bilateral pleural effusions and significant ascites (Fig. 2), with fluid depths up to 9.4 cm in the right lower quadrant, approximately 1.5 cm from the body surface.

### 2.3. Diagnostic process and treatment course

Based on the patient’s clinical presentation and comprehensive evaluation, a diagnosis of overlap syndrome (SSc and SLE) was established. The patient met the 2012 SLICC classification criteria for SLE, and she fulfilled 3 clinical criteria (arthritis, leukopenia, and thrombocytopenia) and 2 immunological criteria (positive ANA and anti-dsDNA antibodies). Additionally, she scored 19 points on the 2013 EULAR classification criteria for SSc, including skin thickening of fingers extending proximal to the metacarpophalangeal (MCP) joints (9 points), pulmonary arterial hypertension (PAH) (2 points), interstitial lung disease (2 points), Raynaud phenomenon (3 points), and positive anti-SCL-70 antibodies (3 points).

During this admission for massive ascites, the patient underwent diagnostic paracentesis and peritoneal drainage for symptom relief. Multiple ascitic fluid analyses showed no evidence of malignancy, chronic liver disease, or infection. Further evaluation with abdominal ultrasound and enhanced magnetic resonance imaging revealed portal vein dilation with splenorenal shunting, suggesting portal collateral circulation. Portal vein flow velocity was 32 cm/s with branch velocity of 17 cm/s.

Following multidisciplinary consultation, the patient was diagnosed with PPMS based on persistent pleural effusions, ascites, and elevated serum CA-125 levels. The condition was attributed to both PAH medications and active underlying disease. Management included gradual discontinuation of Ambrisentan and Sildenafil, along with intensified immunosuppressive therapy. Based on a Systemic Lupus Erythematosus Disease Activity Index 2000 (SLEDAI-2K) score of 8, treatment was escalated to intravenous methylprednisolone 80 mg/d, oral tacrolimus, and belimumab for rapid disease control.

**Figure 3.**
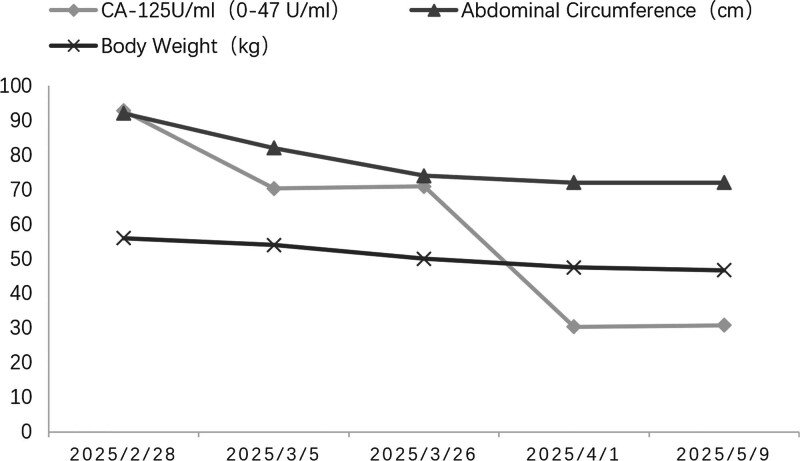
Changes in CA-125 level, abdominal circumference, and body weight before and after treatment. CA-125 = cancer antigen-125.

**Figure 4.**
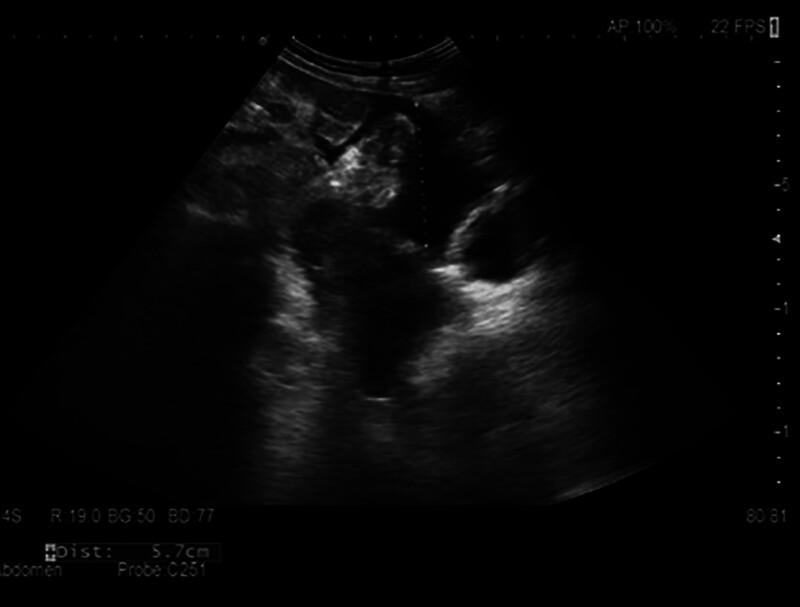
Follow-up ultrasound showing reduction in ascites with maximum depth of approximately 5.7 cm.

Concurrent diuretic therapy with spironolactone and tolvaptan was initiated. The patient showed significant improvement with reduction in waist circumference and body weight. Serum CA-125 levels normalized (Fig. 3), and follow-up imaging at 3 months postdischarge demonstrated marked reduction in ascites (Fig. 4).

## 3. Discussion

PPMS was first described by Tjalma et al in 2005, when they reported a case of an SLE patient presenting with massive ascites and elevated CA-125 levels. Despite undergoing gynecological surgery to rule out malignancy, the patient’s CA-125 levels remained elevated.^[2]^ Another notable case involved an SLE patient who exhibited similar symptoms but showed improvement with medical management alone, suggesting that lupus activity rather than malignancy, was responsible for the clinical presentation. Subsequently, this clinical entity was termed PPMS.^[3]^ This initial description has been pivotal in helping clinicians recognize and understand this rare complication of autoimmune diseases. The term “pseudo-pseudo” was chosen to distinguish it from both Meigs syndrome (involving benign ovarian tumors) and pseudo-Meigs syndrome (involving other pelvic tumors), emphasizing its unique association with autoimmune conditions rather than neoplastic processes.

The precise mechanisms underlying massive ascites and elevated CA-125 levels in PPMS remain incompletely understood. Studies on Meigs syndrome have shown that tumor-induced peritoneal irritation leads to increased CA-125 production by peritoneal mesothelial cells.^[1,2]^ Similarly, in the context of autoimmune diseases, disease activity triggers a cascade of pathological processes. These processes include complement activation, immune complex deposition, vasculitis, and thrombotic injury, which collectively result in elevated levels of various cytokines. These inflammatory mediators may subsequently stimulate peritoneal cells to express CA-125.^[4]^ The inflammatory environment created by active autoimmune disease appears to drive both the formation of effusions and the elevation of CA-125, although the exact pathways remain to be fully elucidated.

The diagnosis of PPMS requires careful exclusion of other conditions with lupus-related serositis. Literature review indicates that lupus patients with nephritis, peritonitis, lupus-associated protein-losing enteropathy, or pelvic infections such as tuberculosis may present with ascites or peritonitis.^[5,6]^ However, in these conditions, CA-125 level elevation is uncommon and typically is only marginally above normal levels. Li^[7]^ reported 3 cases of PPMS that involved lupus nephritis and hypoalbuminemia, both known pathological factors for causing ascites and pleural effusions. However, the markedly elevated CA-125 levels in these cases could not be explained by nephritis and hypoalbuminemia alone, leading to a diagnosis of PPMS. Notably, A retrospective analysis of PPMS cases revealed that only one patient was male,^[8]^ suggesting that male individuals are rarely affected by this disease entity. This observation supports the hypothesis on a potential role of female reproductive organs – such as the ovaries and adnexa – and their associated mesothelial cells in the high expression of CA-125. Furthermore, most reported cases demonstrated low complement levels, particularly complement C3, suggesting that C3-containing immune complexes may play a significant role in the progression of PPMS.^[7]^

Additionally, the combined use of pulmonary arterial pressure-lowering medications may enhance peripheral arterial vasodilation and increase capillary-venous pressure, including in collateral vessels.^[9]^ We hypothesized that the concurrent administration of Ambrisentan and Sildenafil in patients with peripheral venous occlusive disease may lead to pulmonary edema and elevated hepatic venous pressure, ultimately resulting in ascites development. Therefore, once pulmonary arterial pressure was stabilized, we gradually discontinued medications that could potentially cause peripheral arterial vasodilation and increase cardiac output, specifically Ambrisentan and Sildenafil. The introduction of Spironolactone and Tolvaptan helped reduce posterior circulation pressure and gradually improved collateral blood flow. This therapeutic approach suggests that managing hemodynamic changes through careful medication adjustment may be effective in controlling ascites secondary to altered cardiac function.

In conclusion, this case report underscores the complex intricate nature of autoimmune diseases, multi-organ disorders that can present with various complications and comorbidities. Our patient with overlap syndrome exemplifies the multiple pathophysiological mechanisms and complex interactions that can occur in these conditions, particularly when complicated by PPMS. PPMS represents a rare but significant complication of autoimmune diseases that warrants special attention. Patients presenting with massive ascites and elevated CA-125 levels may be mistakenly diagnosed with Meigs syndrome or pseudo-Meigs syndrome associated with benign or malignant ovarian tumors. This case highlights 2 crucial contributing factors in PPMS development: inadequate control of the underlying autoimmune disease and potential medication-induced effects. The diagnostic journey of this patient offers valuable lessons for clinical practice. First, it emphasizes the importance of maintaining a broad differential diagnosis when encountering patients with massive pleural effusions, ascites, and elevated CA-125 levels. Second, it demonstrates the need for careful consideration of medication effects, particularly in complex cases involving multiple therapeutic agents. Additionally, in terms of the prognosis, individual case reports have indicated that patients diagnosed with PPMS at an early stage, including those who experienced sustained remission, may eventually relapse and be diagnosed with conditions such as diffuse large B-cell lymphoma.^[10]^ This finding underscores the importance of regular imaging surveillance for patients with ambiguous immunophenotypes and atypical clinical presentations, in order to further exclude underlying malignant tumors. Finally, this case reinforces the critical role of comprehensive evaluation and multidisciplinary approach in managing such complex presentations. The successful outcome achieved through careful medication adjustment and targeted immunosuppressive therapy underscores the importance of addressing both the underlying autoimmune condition and its complications. For clinicians, this case serves as a reminder that PPMS should be included in the differential diagnosis when encountering patients with autoimmune diseases who present with unexplained serositis and elevated CA-125 levels. Early recognition of this disease entity can prevent unnecessary invasive procedures and guide appropriate therapeutic interventions. This understanding may lead to more targeted and effective treatment strategies for similar cases in the future.

The lessons learned from this case contribute to our growing knowledge of the complex interactions among autoimmune diseases, their complications, and therapeutic interventions, ultimately helping to improve patient care in this challenging clinical setting.

## Author contributions

**Conceptualization:** Hao Zhang, Bingbing Dai.

**Data curation:** Jintao Zhang, Ningning Li.

**Project administration:** Bingbing Dai.

**Supervision:** Bingbing Dai.

**Writing – original draft:** Hao Zhang.

**Writing – review & editing:** Hao Zhang, Jintao Zhang.
